# Insight into the Gas-Induced Phase Transformations in a 2D Switching Coordination Network via Coincident Gas Sorption and *In Situ* PXRD

**DOI:** 10.1021/acsmaterialslett.3c01520

**Published:** 2024-01-23

**Authors:** Shi-Qiang Wang, Volodymyr Bon, Shaza Darwish, Shao-Min Wang, Qing-Yuan Yang, Zhengtao Xu, Stefan Kaskel, Michael J. Zaworotko

**Affiliations:** ‡ Institute of Materials Research and Engineering (IMRE), Agency for Science, Technology and Research (A*STAR), 2 Fusionopolis Way, Singapore 138634, Republic of Singapore; § Faculty of Chemistry, Technische Universität Dresden, Bergstrasse 66, Dresden 01062, Germany; ⊥ Bernal Institute, Department of Chemical Sciences, University of Limerick, Limerick V94 T9PX, Ireland; ∥ School of Chemical Engineering and Technology, Xi’an Jiaotong University, Xi’an 710049, China

## Abstract

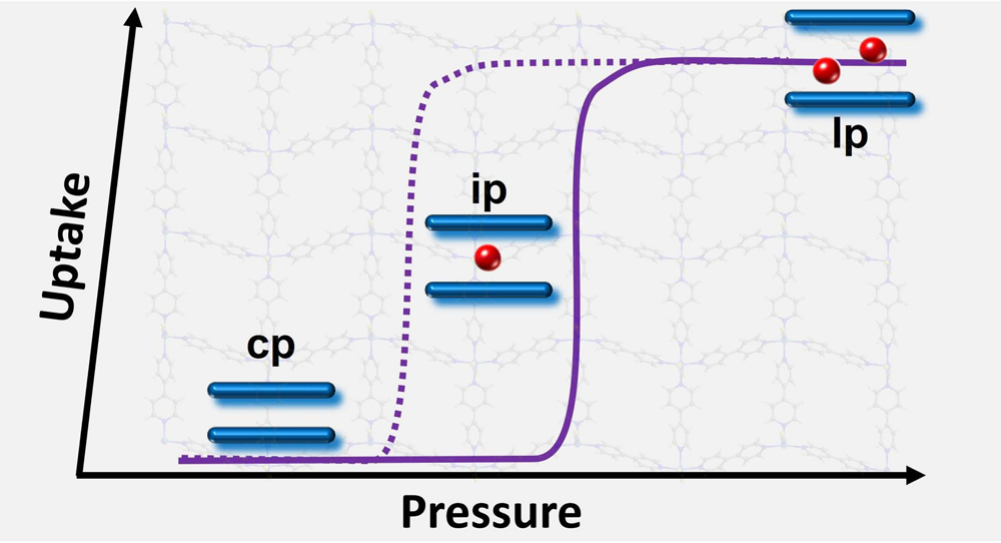

Switching coordination networks (CNs) that reversibly transform between narrow or closed pore (cp) and large pore (lp) phases, though fewer than their rigid counterparts, offer opportunities for sorption-related applications. However, their structural transformations and switching mechanisms remain underexplored at the molecular level. In this study, we conducted a systematic investigation into a 2D switching CN, [Ni(bpy)_2_(NCS)_2_]_n_, sql-1-Ni-NCS (1 = bpy = 4,4′-bipyridine), using coincident gas sorption and *in situ* powder X-ray diffraction (PXRD) under low-temperature conditions. Gas adsorption measurements revealed that C_2_H_4_ (169 K) and C_2_H_6_ (185 K) exhibited single-step type F–IV^s^ sorption isotherms with sorption uptakes of around 180–185 cm^3^ g^–1^, equivalent to four sorbate molecules per formula unit. Furthermore, parallel *in situ* PXRD experiments provided insight into sorbate-dependent phase switching during the sorption process. Specifically, CO_2_ sorption induced single-step phase switching (path I) solely between cp and lp phases consistent with the observed single-step type F–IV^s^ sorption isotherm. By contrast, intermediate pore (ip) phases emerged during C_2_H_4_ and C_2_H_6_ desorption as well as C_3_H_6_ adsorption, although they remained undetectable in the sorption isotherms. To our knowledge, such a cp-lp-ip-cp transformation (path II) induced by C_2_H_4/6_ and accompanied by single-step type F–IV^s^ sorption isotherms represents a novel type of phase transition mechanism in switching CNs. By virtue of Rietveld refinements and molecular simulations, we elucidated that the phase transformations are governed by cooperative local and global structural changes involving NCS^–^ ligand reorientation, bpy ligand twist and rotation, cavity edge (Ni-bpy-Ni) deformation, and interlayer expansion and sliding.

Flexible metal–organic frameworks (FMOFs) or “third generation” porous coordination polymers/networks (PCPs/PCNs) have attracted increasing attention thanks to their structural flexibility which can be stimulus-induced.^1−7^ This phenomenon could enable applications such as gas storage and separation, to name a few,^8−13^ in the context of the “age of gas”.^14^ A characteristic of FMOFs is that they tend to exhibit S-shaped or “stepped” sorption isotherms concomitant with guest-induced structural transformations.^15−17^ A small yet important and growing subset of FMOFs is switching coordination networks (CNs) that undergo extreme guest-induced structural transformations between their “closed pore”, cp, nonporous and “large pore”, lp, porous phases, thereby featuring such stepped or type F–IV sorption isotherms.^18^ Switching CNs could offer higher working capacity, selectivity, and better thermal management than rigid sorbents with type I sorption isotherms.^19−24^

On the other hand, the structural flexibility of switching CNs also poses challenges for molecular-level structural elucidation that is crucial for understanding the underlying host–guest interactions and switching mechanisms. Switching CNs typically undergo phase transformations between their as-synthesized lp and activated cp phases (path I, Scheme 1a). In some instances, one or more intermediate pore (ip) phases that are partially open can exist during desorption (path II, Scheme 1b), adsorption (path III, Scheme 1c) or both processes (path IV, Scheme 1d). The resulting type F–IV gas sorption isotherms that reflect such phase transformations can be subcategorized into single-step profile (type F–IV^s^), multistep profile (type F–IV^m^), or combinations thereof (type F–IV^sm^: single-step adsorption with multistep desorption; type F–IV^ms^: multistep adsorption with single-step desorption), as illustrated in Scheme 1. Measurement of gas sorption isotherms is therefore a general approach for monitoring and classifying phase transformations in switching CNs, based mainly on the number of sorption steps/plateaus and their corresponding sorption uptakes.

**Scheme 1:**
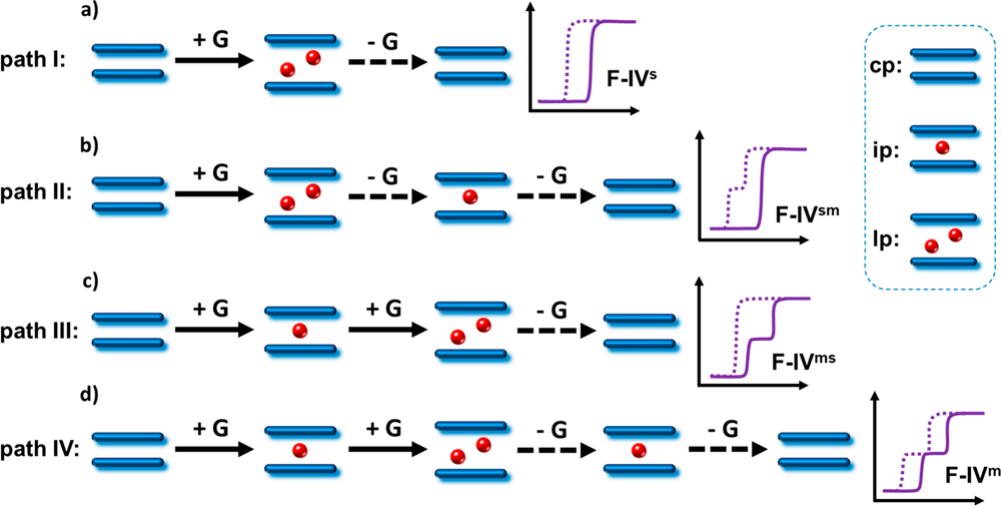
Possible Structural Evolution Paths for 2D Switching CNs Induced by Sorbates/Guest Molecules (G, red balls) with Each Phase Stage Reflected As a Plateau in the Corresponding Type F–IV Sorption Isotherms Solid line/arrow: adsorption process; dashed line/arrow: desorption process.

However, relying solely on sorption isotherms can sometimes yield incomplete or even misleading insight into the phase transformations of switching CNs. This is because their sorption profile can be influenced by various extrinsic factors such as the test temperature/pressure and specific sorbate being studied.^17,18,25^ For example, initial studies on ELM-11 suggested a single-step phase transformation as indicated by CO_2_ sorption isotherms (type F–IV^s^) measured at ambient temperature and pressure.^26^ Nevertheless, further investigation revealed that its full scope of phase transformations includes three sorption steps, as evidenced by CO_2_ sorption data (type F–IV^m^) collected at 195 K or under high pressure (Figure S1a).^27^ For DUT-8(Ni), it is perhaps counterintuitive that C_2_H_6_ and C_2_H_4_ exhibited two-step phase transformation with the formation of **ip** phases while N_2_, CO_2_, and *n*-butane sorption resulted in a single-step transformation, even though their sorption isotherms all exhibit single-step type F–IV^s^ profiles.^28^ These examples demonstrate the complexity of switching mechanisms and underscore a need for advanced *in situ* characterization techniques to monitor the structural evolution of switching CNs during gas sorption.^29^

In this context, integrating gas sorption measurement with *in situ* powder X-ray diffraction (PXRD) is considered one of the most direct approaches to observe structural changes during the entire gas adsorption/desorption process. While gas-loaded PXRD is not uncommon for FMOFs or switching CNs,^26,27,30−41^ it usually has to be guided by *ex situ* gas sorption isotherms measured beforehand. This asynchronous approach may introduce uncertainty regarding the accuracy and consistency. Recent advancements in coincident gas sorption and *in situ* PXRD techniques have effectively addressed this issue as demonstrated by several successful studies,^28,42−47^ thereby enhancing our overall understanding of phase switching mechanisms and facilitating the custom design of the next generation of switching CNs. This prompted us to investigate a prototypical 2D switching CN with a square lattice (sql) topology through coincident gas sorption and *in situ* PXRD, complemented by Rietveld refinements and molecular simulations.

2D sql CNs with general formula [M(L)_2_(A)_2_]_n_ (M = divalent metal cation, L = ditopic linker ligand, A = axial counteranion) are modular from a crystal engineering perspective^48−51^ and exemplify the “node and linker” design strategy proposed by Hoskins and Robson over three decades ago.^52,53^ The first reported sorption study for 2D switching **sql** CNs was conducted on [Cu(bpy)_2_(BF_4_)_2_] (bpy = 4,4′-bipyridine), ELM-11, which was observed to exhibit single (type F–IV^s^) or multistep (type F–IV^m^) sorption isotherms (Scheme 1a, d) induced by gases such as N_2_, Ar, CO_2,_ C_2_H_2_ and *n*-butane.^26,27,33,43,54−58^ Recently, we studied the sorption properties of three previously known **sql** CNs [M(bpy)_2_(NCS)_2_]_n_ (M = Fe, Co, or Ni),^59−65^ sql-1-M-NCS, which are closely related to the ELM platform. CO_2_ sorption for sql-1-M-NCS resulted in single-step type F–IV^s^ isotherms (Scheme 1a) and the switching pressures were found to be metal-ion controlled with the Ni version being the “softest”.^59,61^ It was later reported that sql-1-Ni-NCS exhibited even lower switching pressure and higher sorption uptake for C_2_H_2_ than for CO_2_.^62^

These findings prompted us to further study the adsorbate effect of nine gases on sql-1-Ni-NCS (Figure S2).^64^ It was observed that the sorption of C_2_H_4_ and C_2_H_6_ at 195 K exhibited switching behavior, but their uptakes did not reach saturation even at 113 kPa. In addition, sorption of C_3_H_6_ (propylene) and C_3_H_8_ (propane) at 273 K showed negligible uptake, while the C_3_H_4_ (propyne) sorption exhibited a rare type of F–IV^sm^ sorption isotherm (Scheme 1b) with a saturation uptake of 138 cm^3^ g^–1^ that matches its CO_2_ uptake. We anticipated that lower temperature could favor C_2_H_4/6_ and C_3_H_6/8_ sorption,^66,67^ and through coincident gas sorption and *in situ* PXRD, we aim herein to address several open questions: (a) whether C_2_H_4_ and C_2_H_6_ sorption can reach saturation; (b) whether C_3_H_6_ and C_3_H_8_ can induce the phase switching; (c) whether intermediate phases exist during the structural transitions; (d) last, but not least, how the host structure responds to the different gas molecules, i.e., the underlying switching mechanisms.

sql-1-Ni-NCS was prepared by heating its 1D chain CP precursor, {[Ni(bpy)(NCS)_2_(H_2_O)_2_]·bpy}_n_, that can be obtained by water slurry method (see the Supporting Information for details).^61^ It is sustained by Ni(II) ions coordinated equatorially to two types of bpy linker ligands (half are coplanar, half are twisted) with terminal NCS^–^ ligands occupying the axial positions. The interlayer distance is 4.5 Å (Figure 1a), the shortest among bpy-based sql CNs,^68^ and the effective dimension of the square cavity is approximately 7.5 Å × 7.5 Å (Figure 1b), suitable for accommodating small guest molecules. However, the cavity void is blocked by the interdigitated NCS^–^ ligands and sql-1-Ni-NCS is therefore a cp structure. Thermogravimetric analysis (TGA) and water vapor sorption studies revealed that sql-1-Ni-NCS maintains its thermal stability up to 180 °C and, unlike its hydrophilic analogue ELM-11, sql-1-Ni-NCS is hydrophobic.^61^ It remains stable even after storage for four years, as confirmed by PXRD (Figure S3).

**Figure 1:**
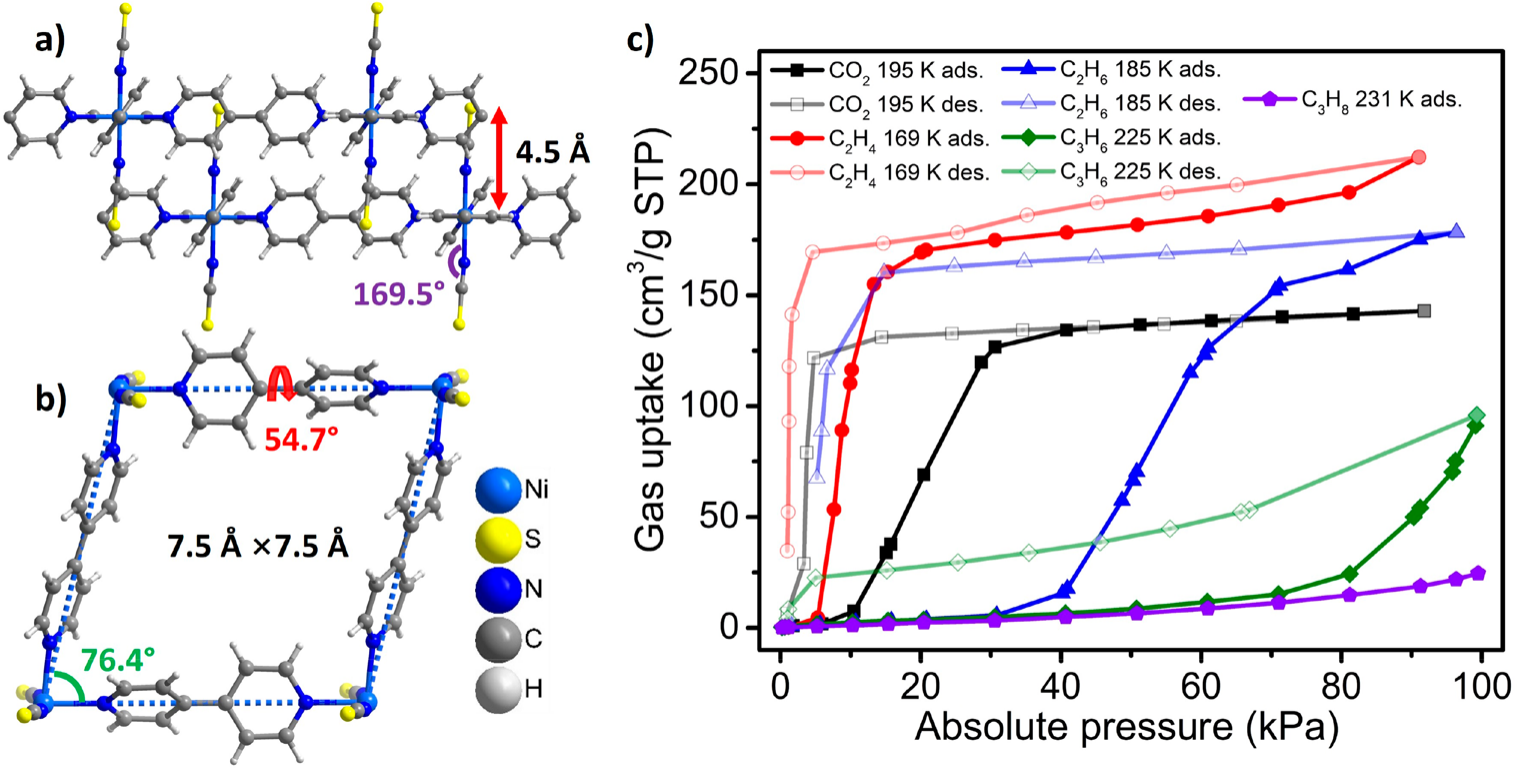
(a, b) Crystal structures of sql-1-Ni-NCS. (c) Gas sorption isotherms for sql-1-Ni-NCS.

For coincident gas sorption and *in situ* PXRD experiments, we investigated five gaseous sorbates on sql-1-Ni-NCS at their sublimation/boiling point temperatures (Figure 1c): CO_2_ (195 K); C_2_H_4_ (169 K); C_2_H_6_ (185 K); C_3_H_6_ (225 K); and C_3_H_8_ (231 K). The 195 K CO_2_ sorption isotherm matched well with our previous results,^61^ verifying the reliability and consistency of the *in situ* gas sorption/PXRD setup used in this study. Interestingly, the 169 K C_2_H_4_ sorption exhibited a similar trend to the previously reported 195 K C_2_H_2_ sorption isotherms (Figure S4).^62^ It plateaued with 185 cm^3^ g^–1^ uptake at around 60 kPa, corresponding to four C_2_H_4_ molecules per formula unit (denoted hereafter as sql-1-Ni-NCS·4C_2_H_4_). Similarly, the 185 K C_2_H_6_ sorption almost reached saturation with around 180 cm^3^ g^–1^ uptake at 95 kPa, corresponding to nearly four C_2_H_6_ molecules per formula unit (denoted hereafter as sql-1-Ni-NCS·4C_2_H_6_). We note that neither C_2_H_4_ (169 K) nor C_2_H_6_ (185 K) sorption is fully desorbed at the final data points of 0.95 and 5.2 kPa, respectively, owing to the sorption program parameters. Nevertheless, our previous study indicates that elevating the temperature to 195 K results in complete desorption of C_2_H_4_ and C_2_H_6_ at around 10 kPa (Figure S2). On the other hand, while the 231 K C_3_H_8_ sorption did not display apparent switching until 1 bar, the 225 K C_3_H_6_ sorption started to switch at around 80 kPa even though it did not reach a plateau at 95 kPa. These results highlight the significant influence of temperature can have on switching CNs, as was the case for ELM-11.^26,27^

The parallel *in situ* PXRD measurements that we conducted provide further insight into the structural evolution of sql-1-Ni-NCS (Figure 2). For example, *in situ* PXRD patterns (Figure 2d) indicate that during CO_2_ adsorption sql-1-Ni-NCS remained as a cp phase until 10 kPa (region: 1–3) and, before reaching the lp phase at around 40 kPa (region: 7–10), it entered a coexisting cp + lp phase between 10–40 kPa (region: 4–6). The CO_2_ desorption branch followed the reverse pathway: lp (region: 10-i-v); coexisting lp + cp (region: vi-vii); cp (region: vii). These profiles suggest that only two phases, cp (sql-1-Ni-NCS) and lp (sql-1-Ni-NCS·3CO_2_), exist during the CO_2_ sorption. In contrast, only two phases, cp (sql-1-Ni-NCS) and lp (sql-1-Ni-NCS·4C_2_H_4/6_), were observed during C_2_H_4_ and C_2_H_6_ adsorption (Figure S5), ip phases appeared during desorption, for example, in region v for C_2_H_4_ and vi for C_2_H_6_ (Figure 2e, f). The ip phase is not directly discernible from the C_2_H_4/6_ desorption branches as no substep plateau was observed in the sorption isotherms. The remaining uptake of around 92 cm^3^ g^–1^ corresponds to two C_2_H_4_ or C_2_H_6_ molecules per formula unit (denoted hereafter as sql-1-Ni-NCS·2C_2_H_4/6_). Furthermore, *in situ* PXRD patterns revealed coexisting cp and ip during C_3_H_6_ adsorption (Figure S6) and that the C_3_H_6_-loaded ip is structurally similar to sql-1-Ni-NCS·2C_2_H_4_/_6_ (Figure S7). Lastly, no structural change was observed during C_3_H_8_ sorption, which is consistent with its minimal uptake (Figure S8).

**Figure 2:**
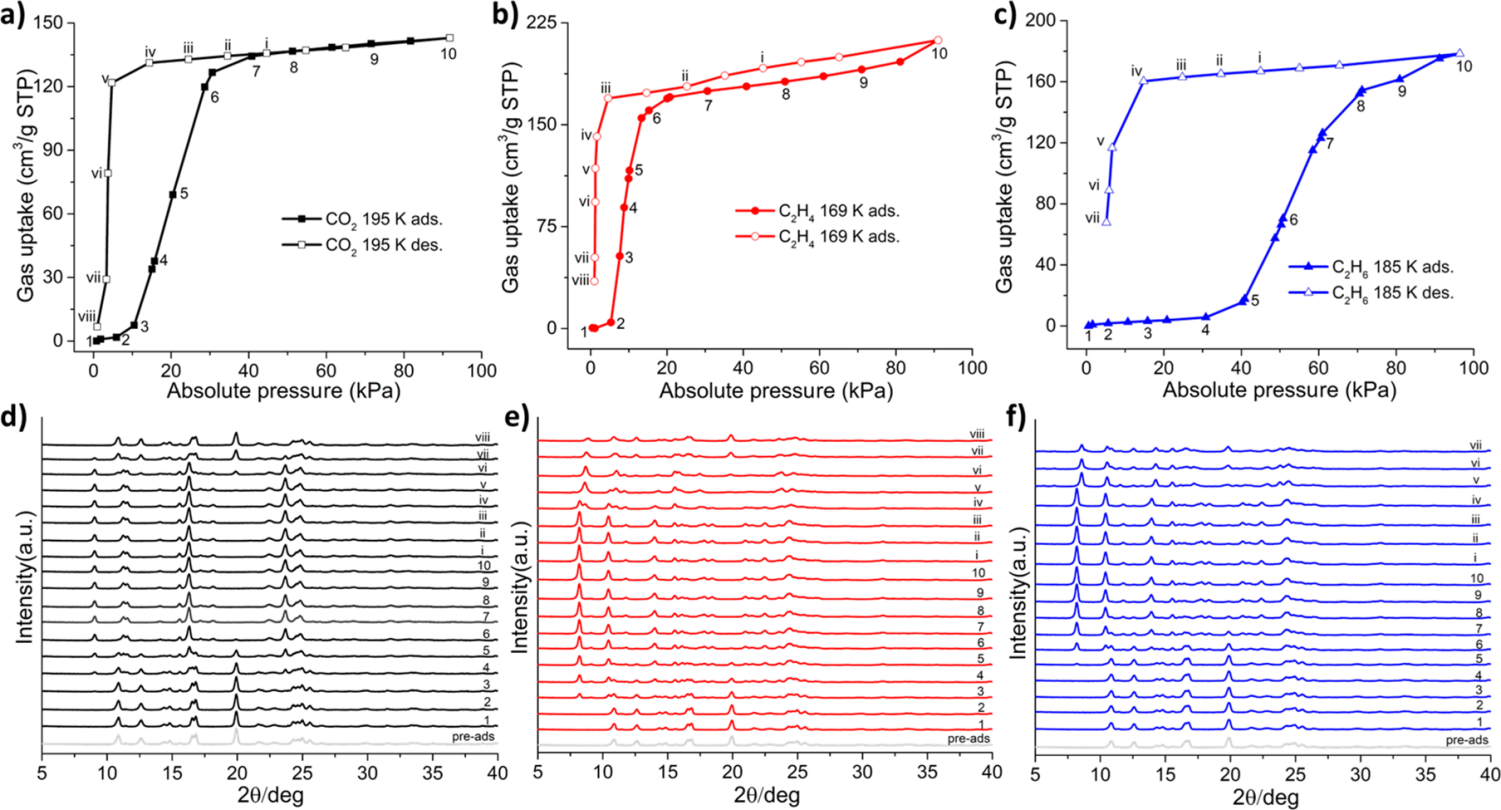
Coincident gas sorption and *in situ* PXRD for (a, d) CO_2_ at 195 K; (b, e) C_2_H_4_ at 169 K; and (c, f) C_2_H_6_ at 185 K.

In order to obtain structural information on sql-1-Ni-NCS during various gas sorption processes, *ab initio* indexing, structural modeling and Rietveld refinement (Figures S9, S10) were applied to determine the crystal structures of sql-1-Ni-NCS·4C_2_H_4/6_ (lp) and sql-1-Ni-NCS·2C_2_H_4/6_ (ip). The crystallographic data reveal that both the C_2_H_4/6_-loaded lp and ip phases maintained the same monoclinic *C*2/*c* space group as the cp and the CO_2_-loaded lp phases (Table S1). While the [NiN_6_] octahedral geometry is consistent for cp, ip, and lp phases, there are variations in the orientation of NCS^–^ ligands toward Ni(II) (∠_Ni–N–CS_ angles) and the torsion angle of the pyridyl rings (Figure 3 and Table S2). The ∠_Ni–N–CS_ angles (Figures 1a and 3a–c) fall within the range of 140.8–169.5° and the bpy torsion angles (Figures 1b and 3d–f) lie between 54.7 and 82.0° for the cp, ip, and lp phases, with angle differences being up to 28.7° and 27.3°, respectively. The dihedral angles between the coplanar bpy ligand and the layer plane (Figure S11) vary from 62.1° in cp to 90.0°, 86.6°, and 87.8° in lp (CO_2_), lp (C_2_H_4/6_), and ip (C_2_H_4/6_), respectively, demonstrating the rotational flexibility of the bpy ligands. The ∠_Ni–Ni–Ni_ angles (Figure 1b and 3d–f) transition from 76.4° (rhombic shape) in cp to around 90.0° (square shape) in ip and lp. The global structural changes reflected at the layer level is that the wavelike fashion of the coplanar bpy ligands (∠_Ni–Ni–Ni_ = 152.8°) in cp transforms into a linear arrangement in the guest-loaded phases, exemplified by the CO_2_-loaded lp (Figure S12). Additionally, the interlayer distances (Figures 1a and 3a–c) increase from 4.5 Å in cp to 5.4 Å in lp (CO_2_), and further to 6.3 Å in lp (C_2_H_4/6_) with a slight reduction to 6.2 Å in ip (C_2_H_4/6_). Such interlayer expansion is accompanied by concurrent layer sliding. For instance, the stacked layers in the cp phase exhibit a shift of 3/8 of the repeating unit, which is reduced to 1/5 in the lp (C_2_H_4/6_) phase (Figure S13). This is primarily attributed to the intercalation of adsorbates into the interlayer space, as suggested by molecular simulation results (Figures S14–17).

**Figure 3:**
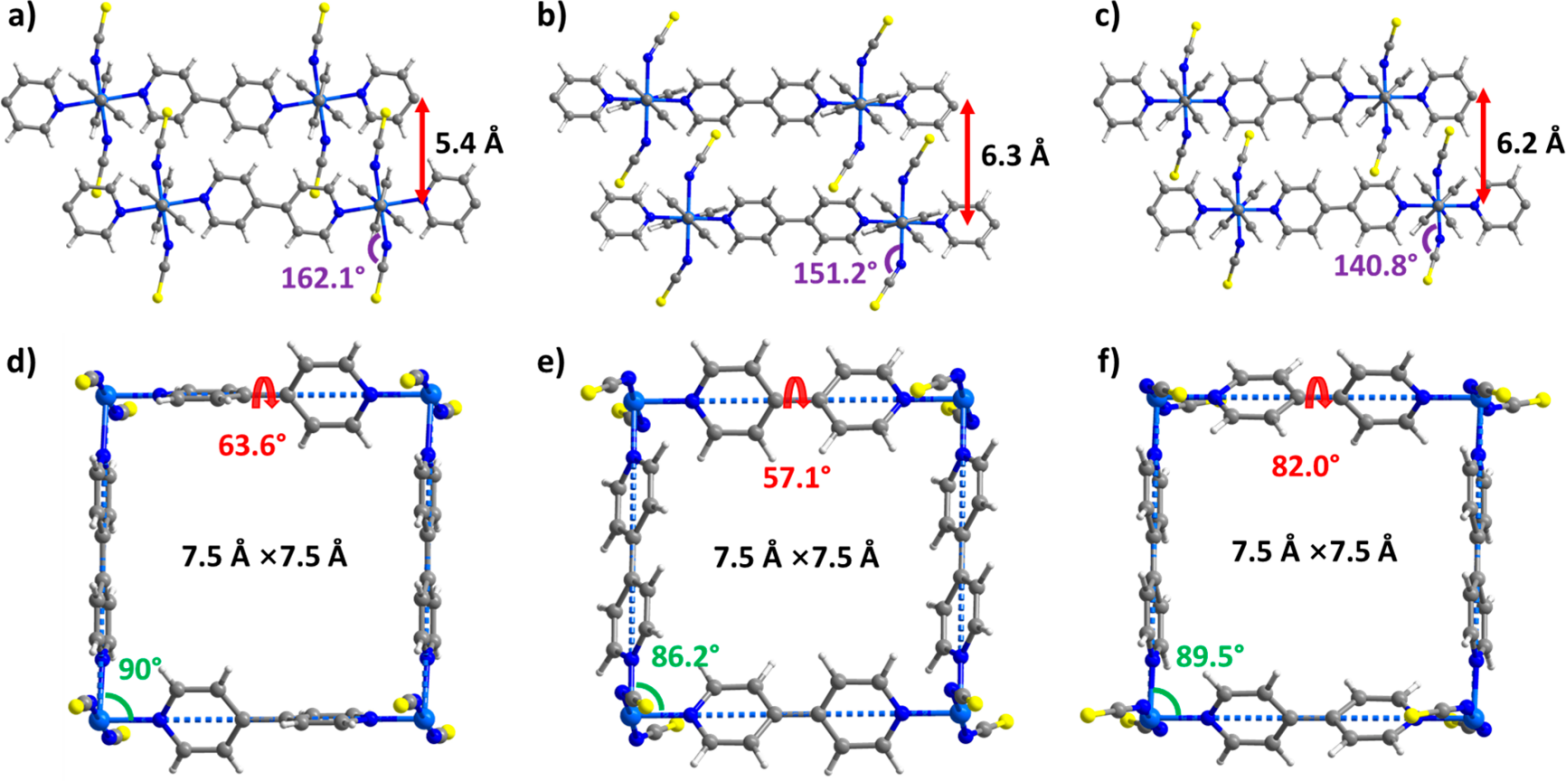
Comparison of crystal structures of *a, d) sql-1-Ni-NCS·3CO_2_ (lp); *b, e) sql-1-NCS 4C_2_H_4/6_ (lp); and *c, f) sql-1-NCS 2C_2_H_4/6_ (ip).

Utilizing both previously reported and newly determined crystal structural information, along with the in situ gas-loaded PXRD data collected in this study, we can understand the changes in PXRD peaks that occur during gas adsorption. For instance, the peak at 2θ = 19.9° is characteristic for the cp phase and originates from the (202) plane of sql-1-Ni-NCS which lies parallel to the network planes (Figure S18a). Consequently, it is understandable that this peak disappears with expansion of the adjacent layers. Additionally, a peak at 2θ = 8.2° emerges in the **lp** phase due to interlayer expansion and sliding. Specifically, it arises from the (002) plane of sql-1-Ni-NCS·4C_2_H_4/6_, intersecting vertically with the bpy ligands (coplanar ones) of each layer (Figure S18b).

Structural comparison of previously reported sql-1-M-NCS·*x*G with a variety of adsorbates (Table S3) reveals the stoichiometric ratio (*x*) of G: M can be 2, 3, or 4 and guest-induced volume expansion ranges from 23.5–114.9%, allowing for classification of five distinct phase types, A to E (Figure 4).^64^ Although *x* is 3 for the CO_2_-loaded phase,^61^ sql-1-Ni-NCS·3CO_2_, it belongs to type A with a low volume expansion (23.5%) comparable to that of a MeOH-loaded phase (sql-1-Fe-NCS·3MeOH) but smaller than that of a CS_2_-loaded phase (sql-1-Fe-NCS·3CS_2_).^69^ This suggests that the adsorbate size can play a role in regulating the degree of interlayer expansion. Whereas C_2_H_2_ shares a similar kinetic diameter and molecular geometry with CO_2_, *x* is 4 for the C_2_H_2_-loaded phase (sql-1-Ni-NCS·4C_2_H_2_).^62^ The volume expansion of sql-1-Ni-NCS·4C_2_H_2_ (40.5%) is larger than that of sql-1-Ni-NCS·3CO_2_ and belongs to type B. C_2_H_4_ and C_2_H_6_ possess kinetic diameters larger than those of C_2_H_2_, enabling sql-1-Ni-NCS·4C_2_H_4/6_ to exhibit even larger volume expansion (44.8%, type C). This volume expansion is relatively small compared to those of C_3_H_6_O (acetone) and CHCl_3_-loaded phases of type D^69,70^ and is much smaller than that of xylene (C_8_H_10_)-loaded phases (type E) which hold the current benchmark for interlayer expansion among bpy-based sql CNs.^60^ With respect to the C_2_H_4/6_-loaded ip, it has almost the same unit-cell volume as the type B structure sql-1-Ni-NCS·4C_2_H_2_.

**Figure 4:**
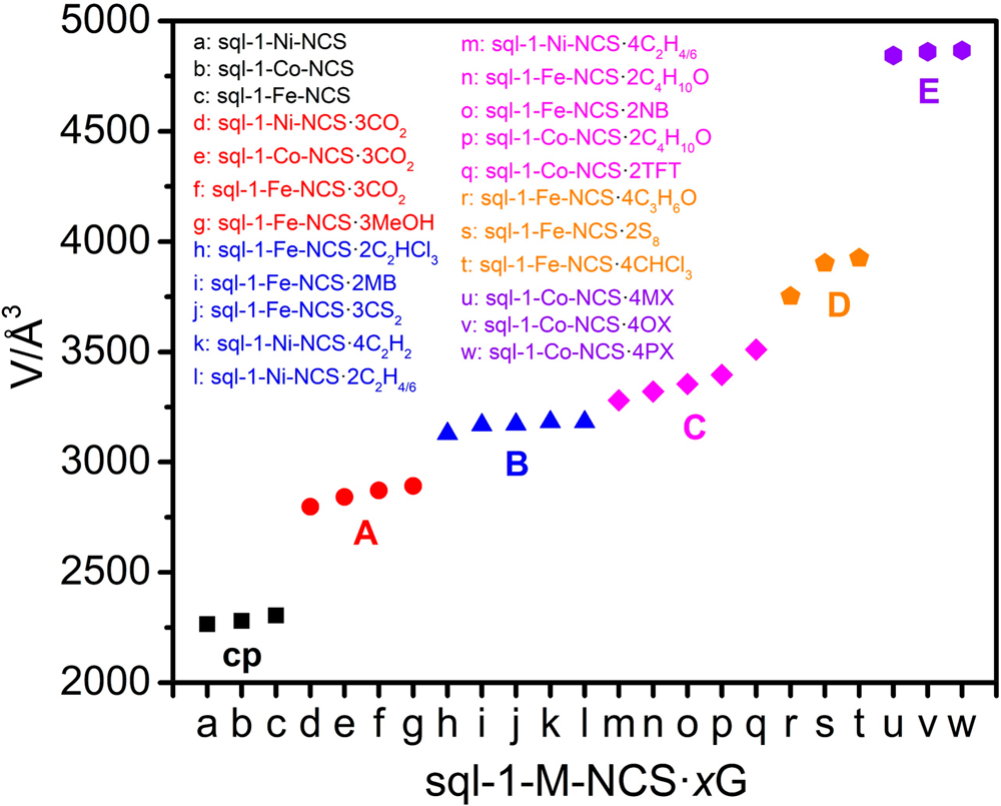
Unit-cell volume (*Z* value normalized to 4) distribution of sql-1-M-NCS·*x*G.

When comparing the gas sorption behaviors of sql-1-Ni-NCS with its analogous ELM-11 (sql-1-Cu-BF_4_), some notable differences are observed despite their comparable cavity sizes (∼7.0–7.5 Å) and interlayer distances (∼4.5 Å). First, sql-1-Ni-NCS displayed significantly higher gate opening pressure than ELM-11 under the same measurement conditions.^27,61^ Second, sql-1-Ni-NCS exhibited only one CO_2_-loaded phase (sql-1-Ni-NCS·3CO_2_) while ELM-11 exhibited three distinct phases: ELM-11·2CO_2_, ELM-11·3CO_2_, and ELM-11·6CO_2_ (Figure S1a).^27^ Additionally, ELM-11 has been reported to have four C_2_H_2_-loaded phases with *x* = 1, 2, 6, 8, respectively (Figure S1b–d).^58^ In terms of structural evolution during gas sorption, prior research revealed that no additional **ip** phase lies between ELM-11 and ELM-11·2CO_2_ or ELM-11·2C_4_H_10_,^43^ despite the observation of ELM-11·1C_2_H_2_ between ELM-11 and ELM-11·2C_2_H_2_ in the C_2_H_2_ sorption isotherms.^57,58^

Gas molecules larger than CO_2_, like *n*-butane (C_4_H_10_), resulted in larger volume expansion for ELM-11 (Table S4) despite having the same ratio (*x* = 2).^43^ This trend aligns with the behavior of sql-1-M-NCS with respect to the sorption of C_2_H_2_, C_2_H_4/6_, C_3_H_6_O, CHCl_3_, and C_8_H_10_ (*x* = 4).^60,62,69,70^ However, it does not necessarily apply to 3D switching CNs such as DUT-8(Ni) and MIL-53(Fe).^28,37^ This discrepancy might be attributed to the degree of dimensional flexibility inherent in FMOFs.^71,72^ The presence of relatively weak interactions, such as van der Waals forces, between adjacent layers in 2D switching CNs implies high flexibility and adaptability. This feature allows 2D switching CNs to accommodate various guest molecules with different sizes and shapes through induced-fit inclusion and claylike intercalation, as illustrated in Figures S19–S23.

In summary, we detail gas-induced phase switching in a 2D sql CN, sql-1-Ni-NCS, using coincident gas sorption and *in situ* PXRD. Thanks to the low temperature gas sorption measurements of C_2_H_4_ (169 K), C_2_H_6_ (185 K), C_3_H_6_ (225 K), and C_3_H_8_ (231 K), we addressed the first two questions outlined earlier: (a) C_2_H_4_ and C_2_H_6_ sorption can reach or nearly reach saturation plateau (185 cm^3^ g^–1^) before 1 bar at their boiling point temperatures, although both sorption isotherms were previously found to be incomplete at 195 K and around 1.1 bar;^64^ (b) C_3_H_6_ can induce partial phase switching while C_3_H_8_ failed to do so before reaching 1 bar at their boiling point temperatures. Subsequently, *in situ* PXRD and Rietveld refinements allowed us to answer the remaining questions: (c) There was no ip phase observed during CO_2_ sorption. However, ip phases were detected during C_2_H_4/6_ desorption and C_3_H_6_ adsorption, although it was not discernible from their respective sorption isotherms; d) In order to accommodate different gas molecules, the structure of sql-1-Ni-NCS undergoes local and global structural changes including NCS ligand reorientation, bpy ligand twist and rotation, cavity edge (Ni-bpy-Ni) deformation, and interlayer expansion.

The discovery of intermediate phases and the structure determination of each phase within sql-1-Ni-NCS emphasize the importance of collecting *in situ* PXRD data throughout the entire gas sorption profile to gain insight into the full landscape of structural transformations.^29,73^ The elucidation of the adsorbate-dependency of the phase transition path and volume expansion could provide useful guidance for utilizing switching CNs in various sorption-related applications, such as gas storage, hydrocarbon separation, and sensing. Given that sql-1-Ni-NCS belongs to a broad family of sql CNs with numerous options for M (metal cation), L (linker ligand), A (counteranion), and G (guest adsorbate),^18^ further sorption studies on such switching CNs are therefore in progress.

## Supporting Information Available

Experimental details, PXRD patterns, and gas sorption isotherms (PDF)

## Supplementary Material

tz3c01520_si_001.pdf

tz3c01520_si_002.cif

tz3c01520_si_003.cif

tz3c01520_si_004.cif

tz3c01520_si_005.cif
