# Supplementary material for: Insight into the Gas-Induced Phase Transformations in a 2D Switching Coordination Network via Coincident Gas Sorption and In Situ PXRD
Source: ACS Mater Lett. 2024 Jan 23;6(2):666–73. doi: 10.1021/acsmaterialslett.3c01520 (PMC10848331; doi:10.1021/acsmaterialslett.3c01520)
Supplement: Supplementary file 1 — tz3c01520_si_001.pdf [file tz3c01520_si_001.pdf]

Supporting Information

Insight into the gas-induced phase transformations in a 2D switching coordination network via coincident gas sorption and in situ PXRD

Shi-Qiang Wang,#a Volodymyr Bon,#b Shaza Darwish,c Shao-Min Wang,d Qing-Yuan Yang,d Zhengtao Xu,*a Stefan Kaskel,*b and Michael J. Zaworotko*c*

[a]*Institute of Materials Research and Engineering (IMRE), Agency for Science, Technology and Research (A*STAR), 2 Fusionopolis Way 138634, Singapore.*

[b]*Faculty of Chemistry, Technische Universität Dresden, Bergstrasse 66, 01062 Dresden, Germany.*

[c]*Bernal Institute, Department of Chemical Sciences, University of Limerick, Limerick V94 T9PX, Ireland.*

[d]*School of Chemical Engineering and Technology, Xi'an Jiaotong University, Xi'an 710049, China.*

**E-mail:* wangsq@imre.a-star.edu.sg; zhengtao@imre.a-star.edu.sg; stefan.kaskel@tu-dresden.de; xtal@ul.ie

#*These authors contributed equally.*

Experimental section

Synthesis of $[Ni(bpy)_2(NCS)_2]_n$ (sql-1-Ni-NCS)

$NiSO_4 \cdot 6H_2O$ (10 mmol, 2.63 g), NaSCN (20 mmol, 1.62 g) and 4,4′-bipyridine (20 mmol, 3.12 g) were added to 50 mL water in a 100 mL bottle. The slurry was stirred continuously for 3 h under room temperature to form the 1D chain CP precursor $\{[Ni(bpy)(NCS)_2(H_2O)_2]\cdot bpy\}_n$ which was then filtered, washed with water and air-dried (yield ~ 95 %). The precursor powder was activated at 60 °C in vacuo for 5 h to transform to the targeted **sql** coordination network: sql-1-Ni-NCS.

Powder X-ray Diffraction (PXRD)

Powder X-ray diffraction experiments were conducted on a Panalytical Empyrean diffractometer (40 kV, 40 mA, Cu $K\alpha_{1,2}$, $\lambda = 1.5418$ Å) at room temperature with a range of $5^{\circ} < 2\theta < 40^{\circ}$.

Coincident gas sorption and *in situ* powder X-ray diffraction measurements

In situ PXRD patterns, measured in parallel to gas physisorption, were collected using a customized Empyrean (Malvern Panalytical GmbH) powder X-ray diffractometer (ω–2θ goniometer, Alpha1 system), equipped with ARS DE-102 closed-cycle helium cryostat. Customized X-ray transparent adsorption cell is based on 1.33″ CF-flange, one part of which is mounted on the cryostat and another is brazed to a beryllium dome. The cell was connected to the low-pressure port of the BELSORP-max (Mictrotrac MRB) volumetric adsorption instrument using 1/8″ stainless steel capillary. The TTL trigger was used for establishing the communication between BELSORP-max and Data Collector (Empyrean) software and certified the automated measurement of the adsorption isotherm and PXRD patterns in the selected points of the isotherm.

The diffraction experiments were conducted in a transmission geometry (ω–2θ scan mode) in the range of 2θ = 5 - 50°. Parallel beam optic involving hybrid 2xGe(220) monochromator, primary divergence slits (1/4°) and motorized anti-scatter slits (1/4°), 4 mm mask, Solar slits (0.04°) on the incident and diffracted beam optics. Pixcel-3D detector in 1D scanning mode (255 active channels) was used for counting the signal. CO_2 (195 K), C_2H_4 (169 K), C_2H_6 (185 K), C_3H_6 (225 K), and C_3H_8 (231 K) adsorption–desorption isotherms for sql-1-Ni-NCS were measured on 13 mg of the sample and PXRD patterns were *in situ* recorded after equilibration (0.1% of pressure change within 5 min) at selected points of the isotherms.

Rietveld refinements combined with molecular simulations

Indexing of PXRD patterns, structural modelling and Rietveld refinement of the crystal structures were performed in Materials Studio 5.0. The unit cell parameters were obtained by ab initio indexing of PXRD patterns and initial structural model was created using the reported structure of sql-1-Ni-NCS$\cdot 3CO_2$ as the starting template.[1] One or two ethane/ethylene molecules per asymmetric unit were added to the model, followed by Rietveld refinement with energies (energy contribution is 1%). Nickel atoms, phenyl rings of 4,4’-bpy molecule, NCS^- anions and guest molecules were defined as

rigid bodies. The final refinement plot is given in Figures S9-S10, and the unit-cell parameters are listed in Table S1 together with those of previously reported phases. The adsorbate locations were further optimised (Figures S14-17) in Materials studio using the Forcite module (quality: ultra-fine, algorithm: smart; forcefield: universal), with fixed atom Cartesian position of the coordination networks. The final structures were deposited in Cambridge Structural Database (CCDC no: 2311949-2311952).

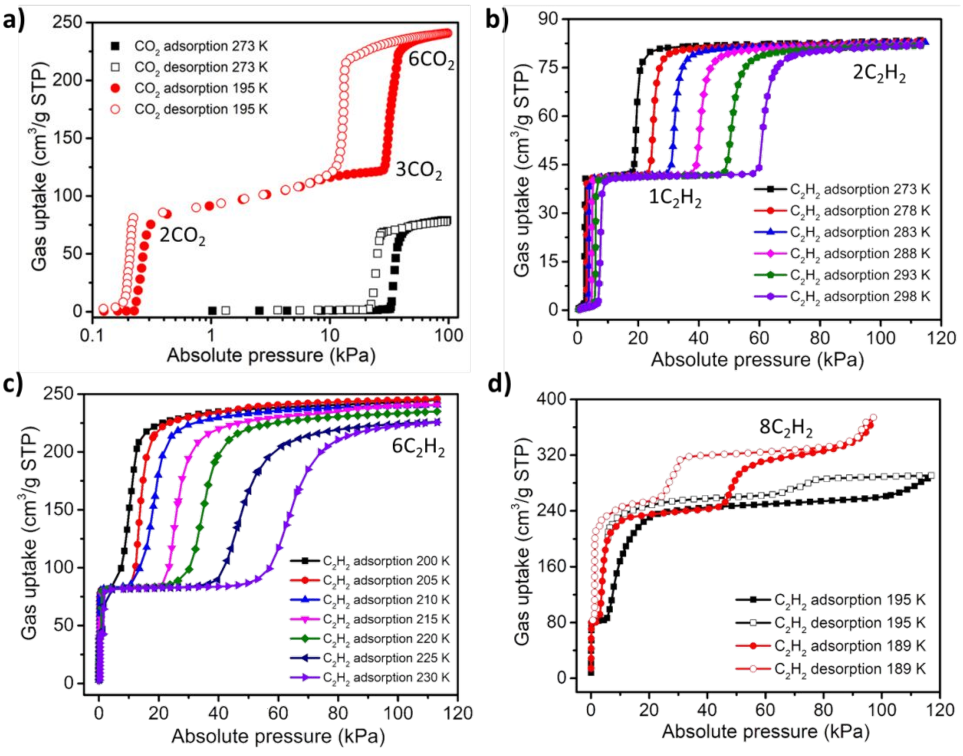

Figure S1. CO_2 and C_2H_2 adsorption isotherms collected at different temperatures for ELM-11 (sql-1-Cu-BF_4) demonstrates the impact of temperature on the phase transformations of ELM-11, with up to three CO_2-loaded phases (2, 3, 6 CO_2 molecules per unit formula) and four C_2H_2-loaded phases (1, 2, 6, 8 C_2H_2 molecules per unit formula) observed at 195 and 189 K, respectively.[2,3]

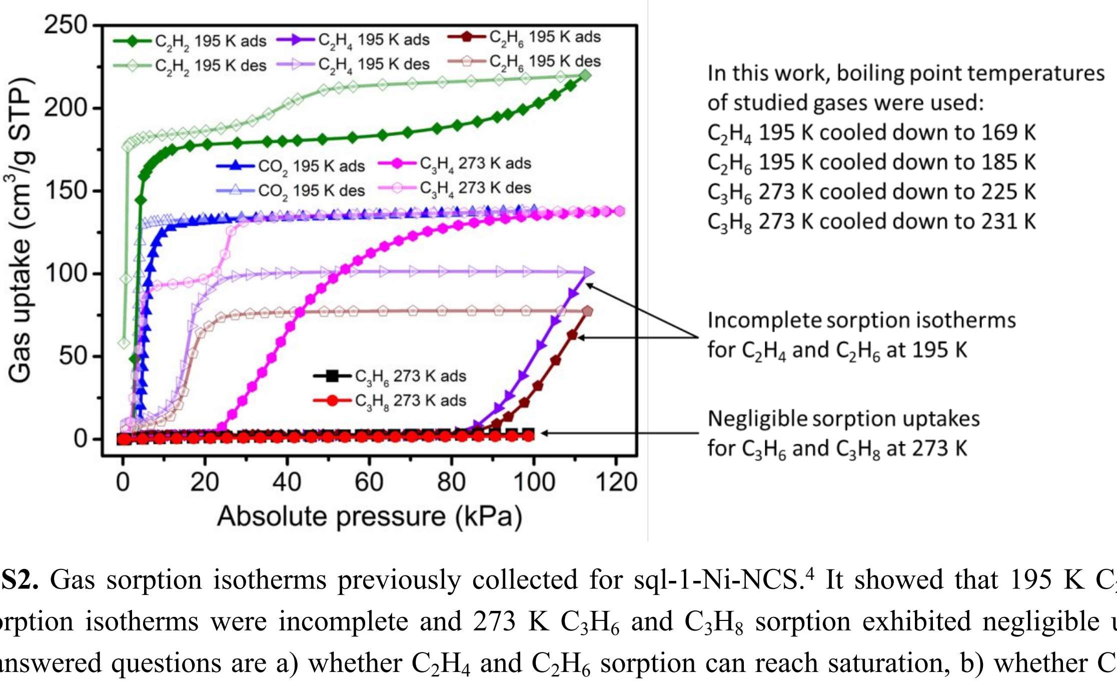

Figure S2. Gas sorption isotherms previously collected for sql-1-Ni-NCS.[4] It showed that 195 K C_2H_4 and C_2H_6 sorption isotherms were incomplete and 273 K C_3H_6 and C_3H_8 sorption exhibited negligible uptakes. The unanswered questions are a) whether C_2H_4 and C_2H_6 sorption can reach saturation, b) whether C_3H_6 and C_3H_8 can induce the phase switching if the measurement temperatures are cooled down to their boiling point temperatures respectively.

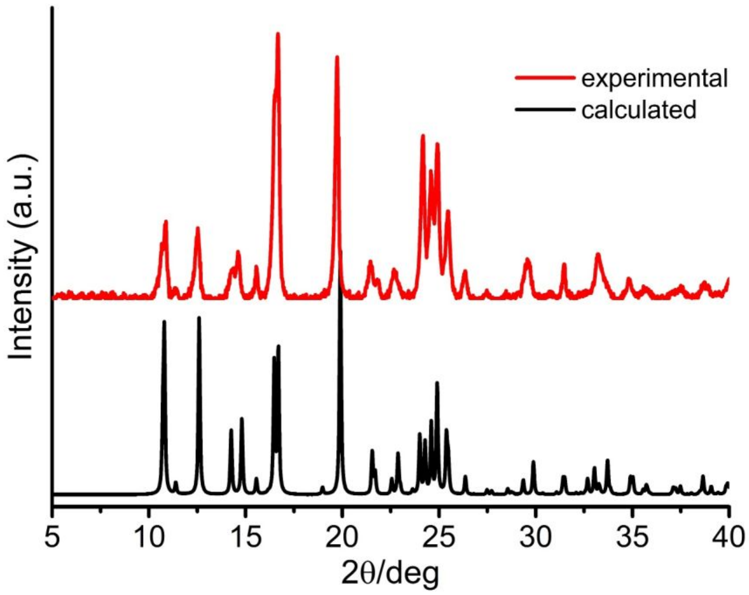

Figure S3. PXRD patterns of sql-1-Ni-NCS after stored in a capped vial for four years.

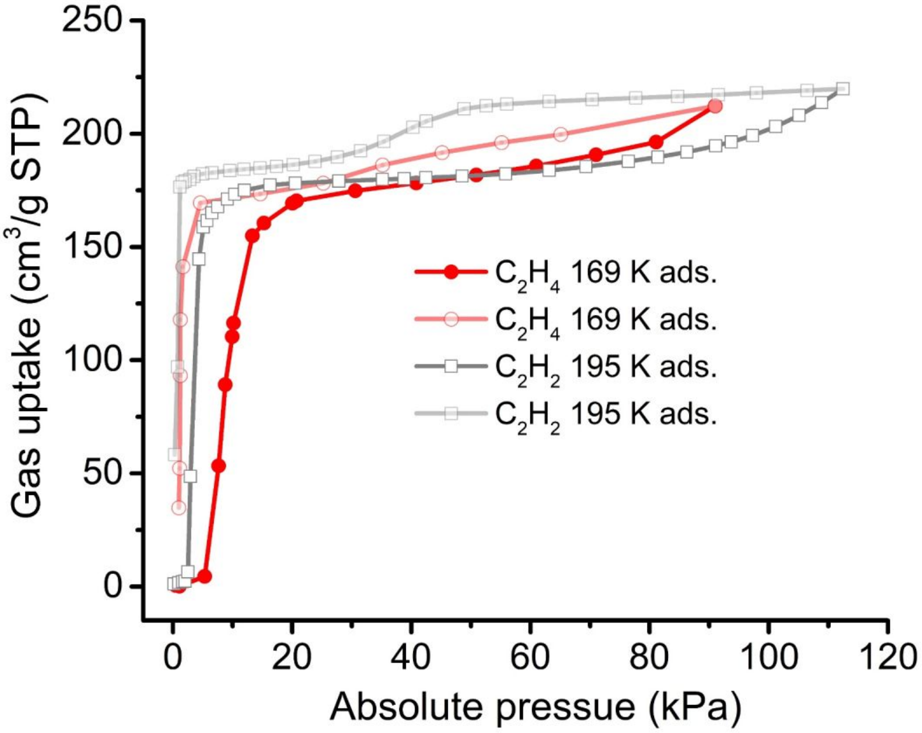

Figure S4. Comparison of the C_2H_4 (169 K) and C_2H_2 (195 K) sorption isotherms for sql-1-Ni-NCS.

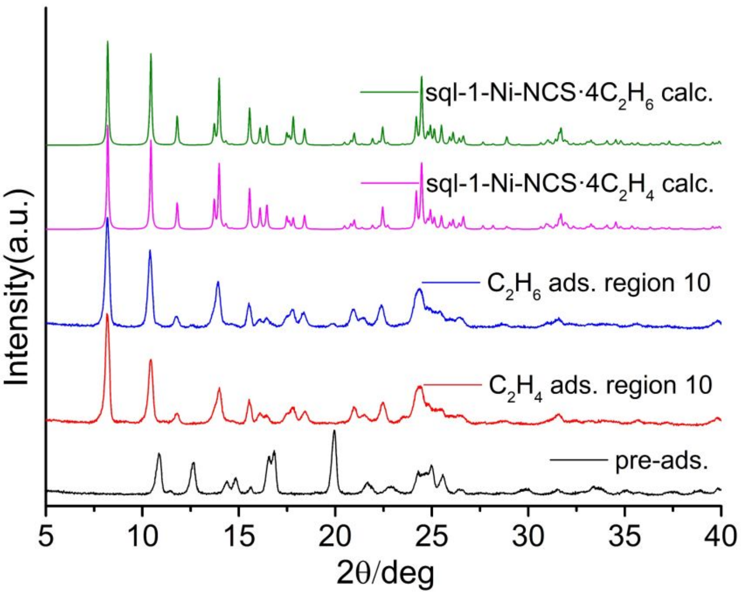

Figure S5. Comparison of the PXRD patterns collected during C_2H_4 and C_2H_6 adsorption at region 10. They match well with the calculated ones derived from the crystal structures (**lp** phases) obtained by Rietveld refinements and molecular simulations.

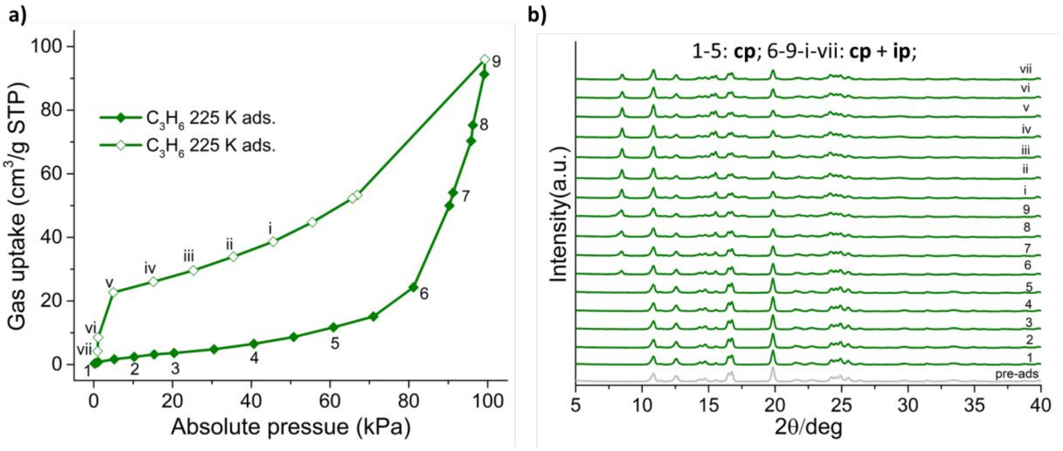

Figure S6. Coincident gas sorption and *in situ* PXRD for C_3H_6 at 225 K.

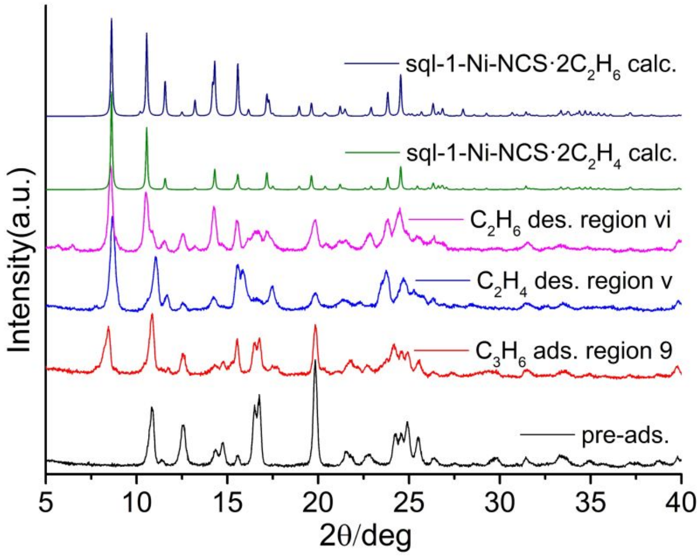

Figure S7. Comparison of the PXRD patterns collected during C_2H_4, C_2H_6, and C_3H_6 sorption at region v, vi and 9, respectively. They are in good agreement with the calculated ones derived from the crystal structures (**ip** phases) obtained by Rietveld refinements and molecular simulations.

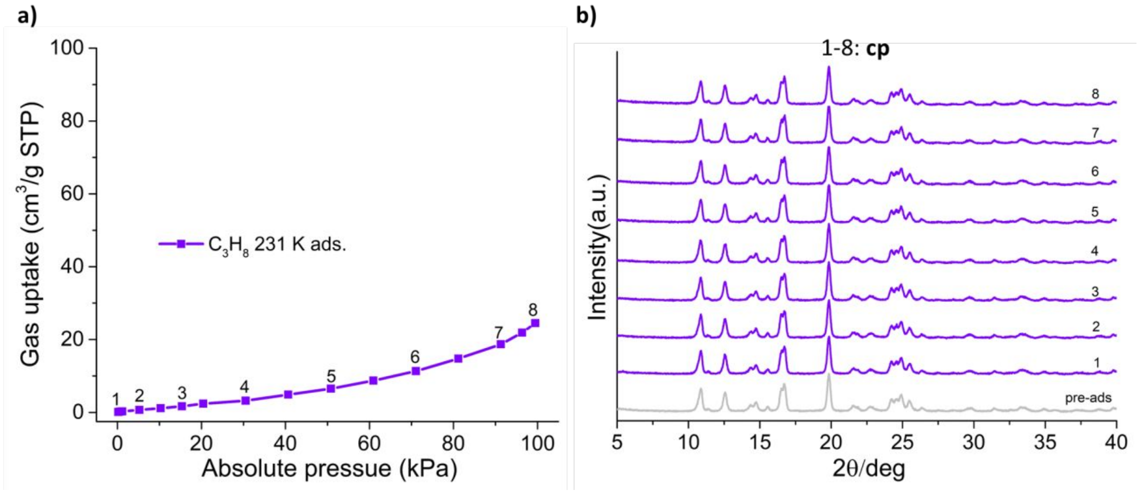

Figure S8. Coincident gas sorption and *in situ* PXRD for C_3H_8 at 231 K.

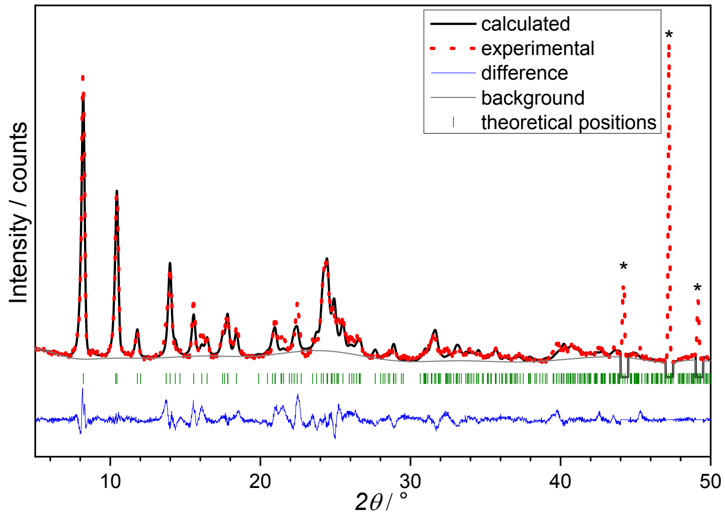

Figure S9. Rietveld refinement (R_p = 0.0920, R_{wp} = 0.1266) of sql-1-Ni-NCS·$4C_2H_{4/6.}$ (The last three peaks marked with asterisks are background peaks originating from the Be-Dome).

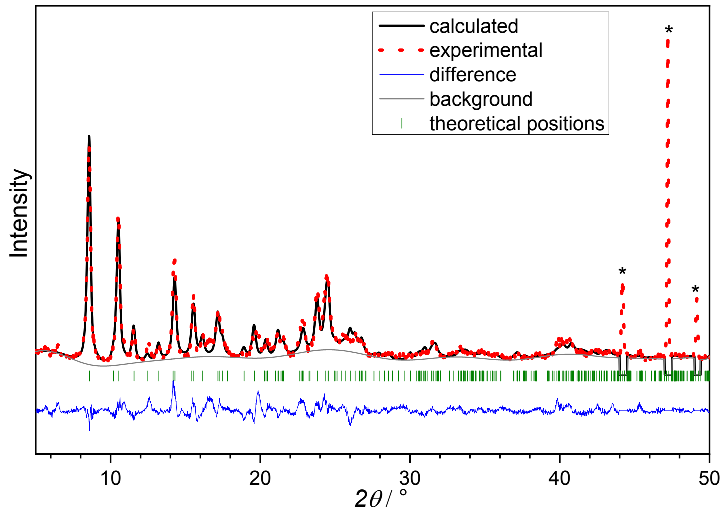

Figure S10. Rietveld refinement (R_p = 0.1008, R_{wp} = 0.1311) of sql-1-Ni-NCS·$2C_2H_{4/6.}$ (The last three peaks marked with asterisks are background peaks originating from the Be-Dome).

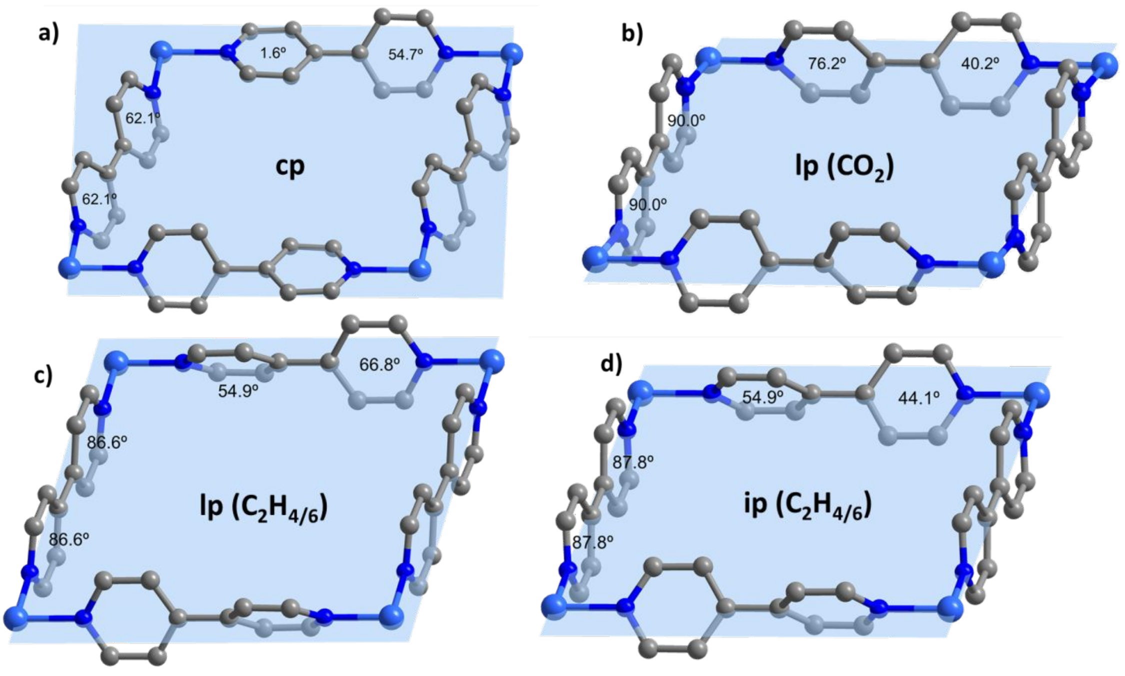

Figure S11. Dihedral angles between the pyridyl rings and the layer plane (Ni-Ni-Ni-Ni) for a) sql-1-Ni-NCS (**cp**), b) sql-1-Ni-NCS·3CO_2 (**lp**), c) sql-1-Ni-NCS·4$C_2H_{4/6}$ (**lp**), and d) sql-1-Ni-NCS·2$C_2H_{4/6}$ (**ip**).

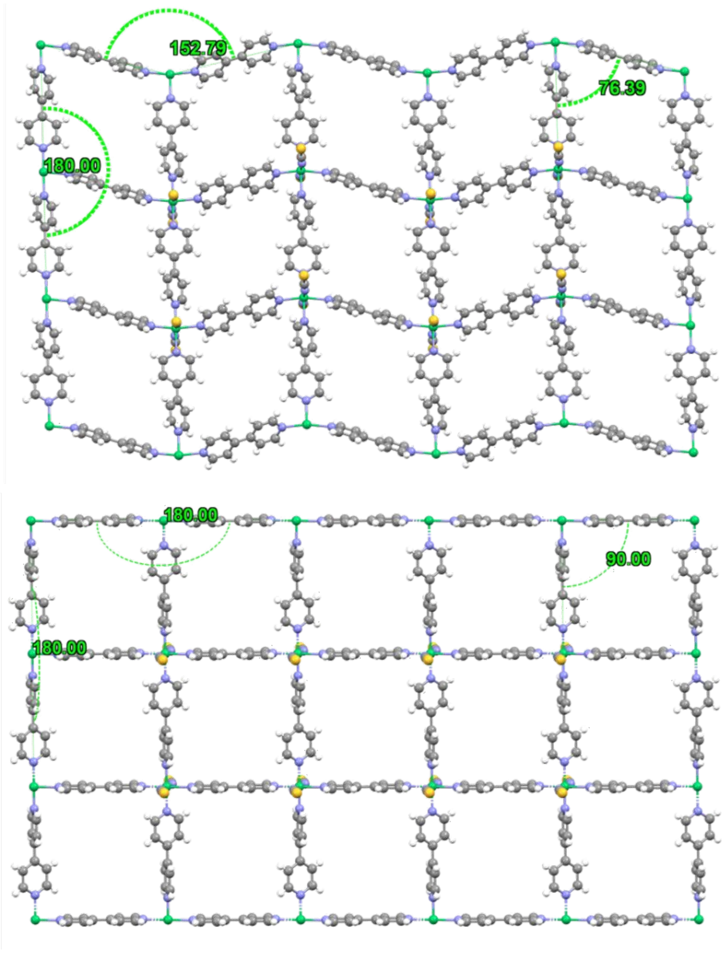

Figure S12. The layer propagation fashion in sql-1-Ni-NCS (top) and sql-1-Ni-NCS·3CO_2 (bottom).

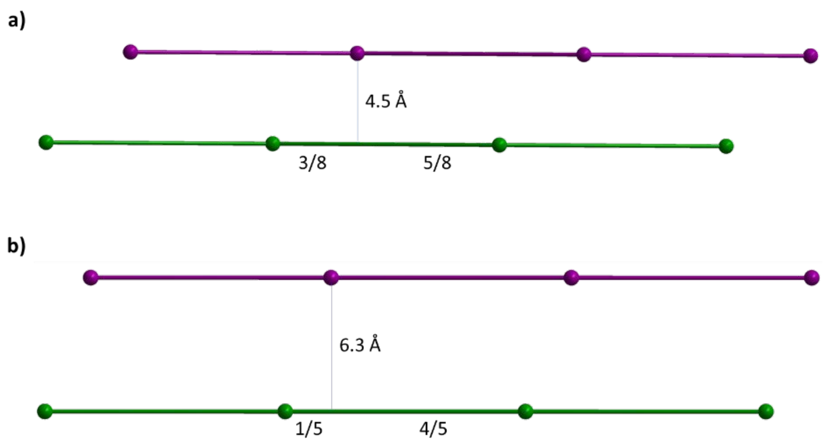

Figure S13. Illustration of the interlayer expansion and sliding phenomenon from the phases of a) sql-1-Ni-NCS (**cp**) to b) sql-1-Ni-NCS·4$C_2H_{4/6}$(**lp**).

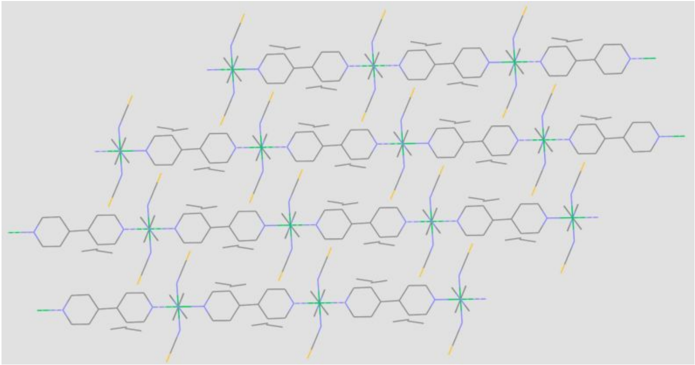

Figure S14. Layer packing fashion (viewed along ***b*** axis) of sql-1-Ni-NCS·4C_2H_4. (Hydrogen atoms are hereafter omitted for clarification.)

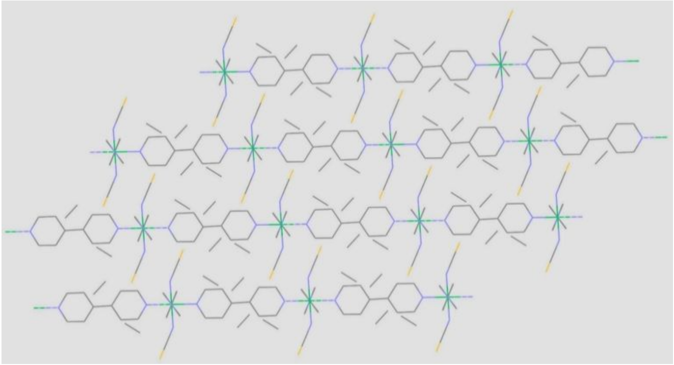

Figure S15. Layer packing fashion (viewed along ***b*** axis) of sql-1-Ni-NCS·4C_2H_6.

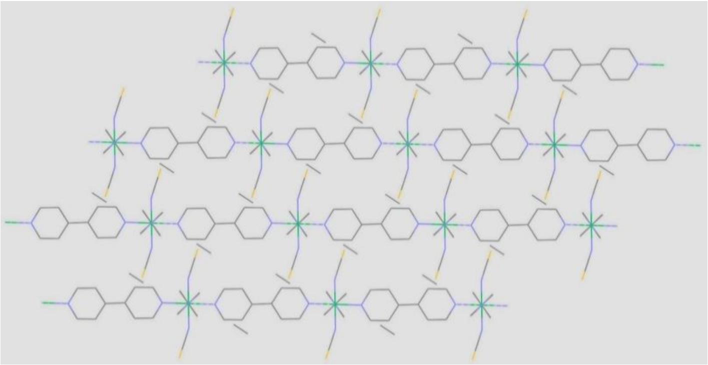

Figure S16. Layer packing fashion (viewed along ***b*** axis) of sql-1-Ni-NCS·2C_2H_4.

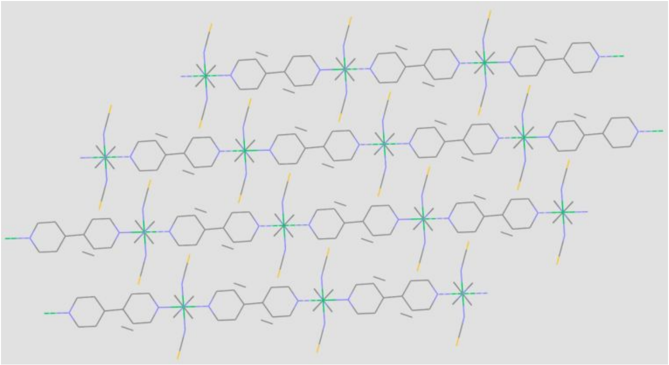

Figure S17. Layer packing fashion (viewed along ***b*** axis) of sql-1-Ni-NCS·2C_2H_6.

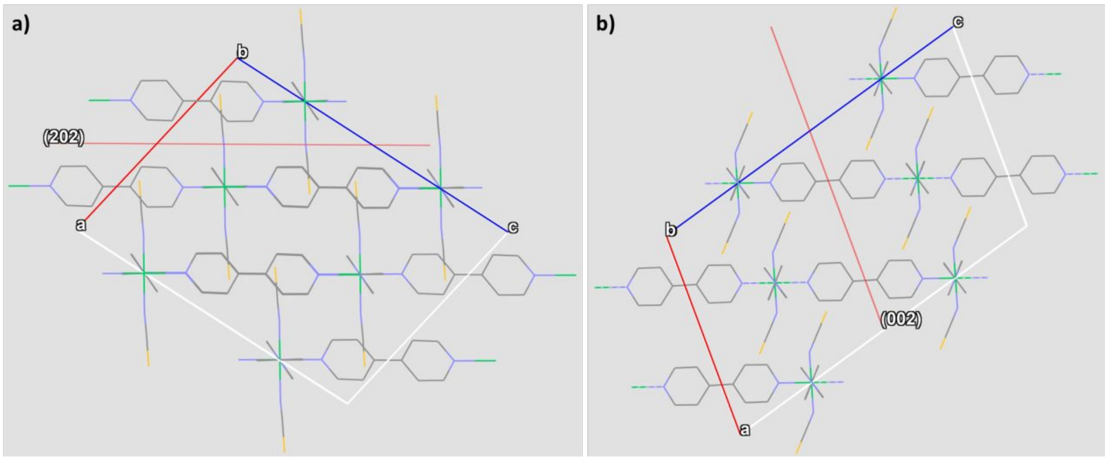

Figure S18. a) (202) plane in the structure of sql-1-Ni-NCS and b) (002) plane in the structure of sql-1-Ni-NCS·4$C_2H_{4/6}$.

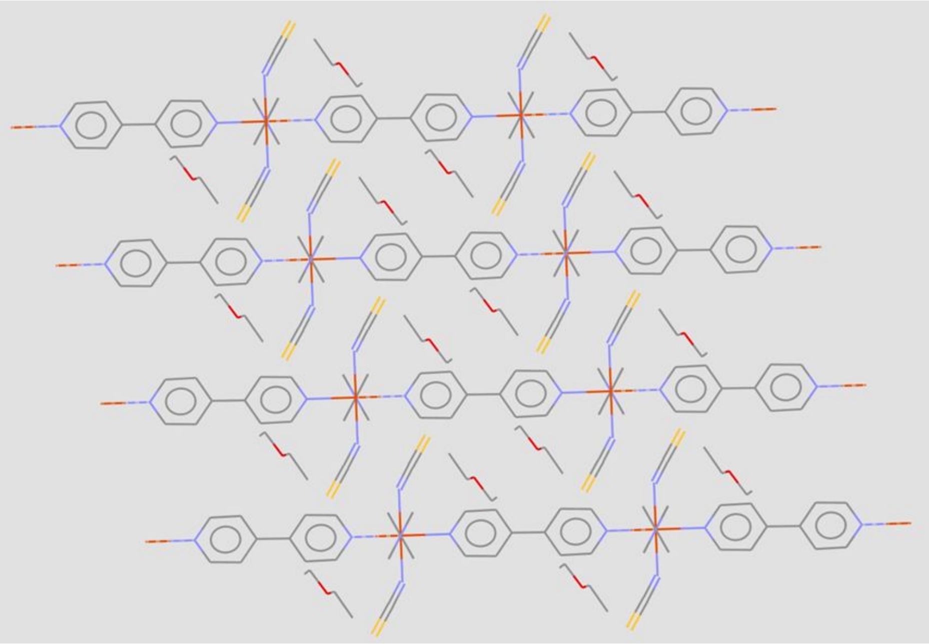

Figure S19. Layer packing fashion (viewed along ***b*** axis) of sql-1-Fe-NCS·2$C_4H_{10}O$ ($C_4H_{10}O$ = diether, refcode: QAGWIG).

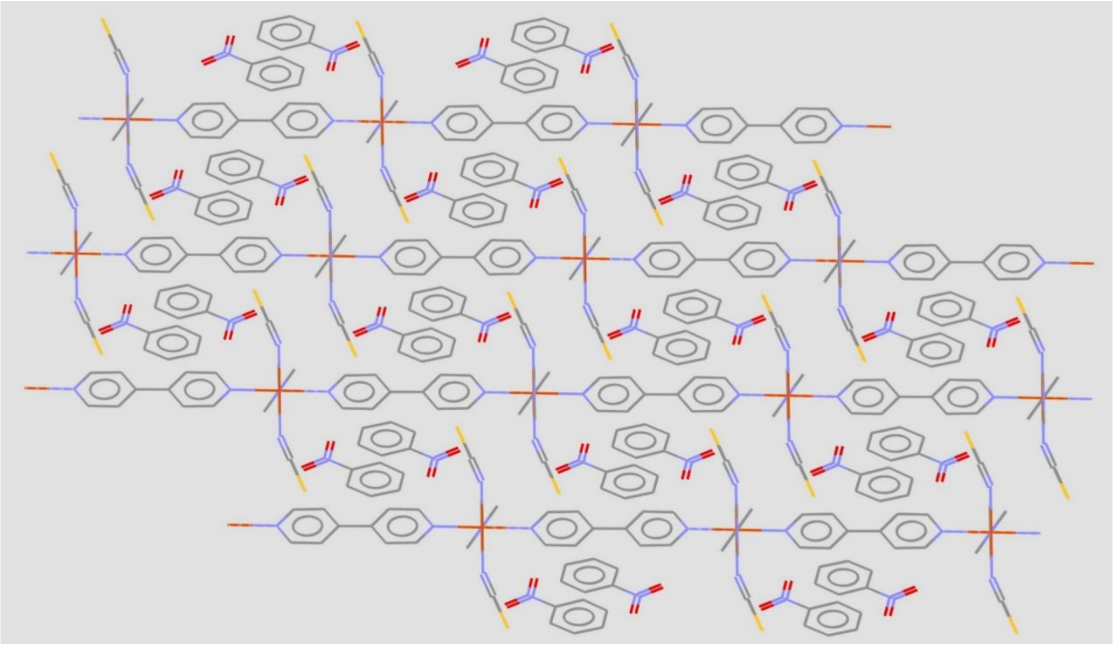

Figure S20. Layer packing fashion (viewed along ***b*** axis) of sql-1-Fe-NCS·2NB (NB = nitrobenzene, refcode: QAGWEC).

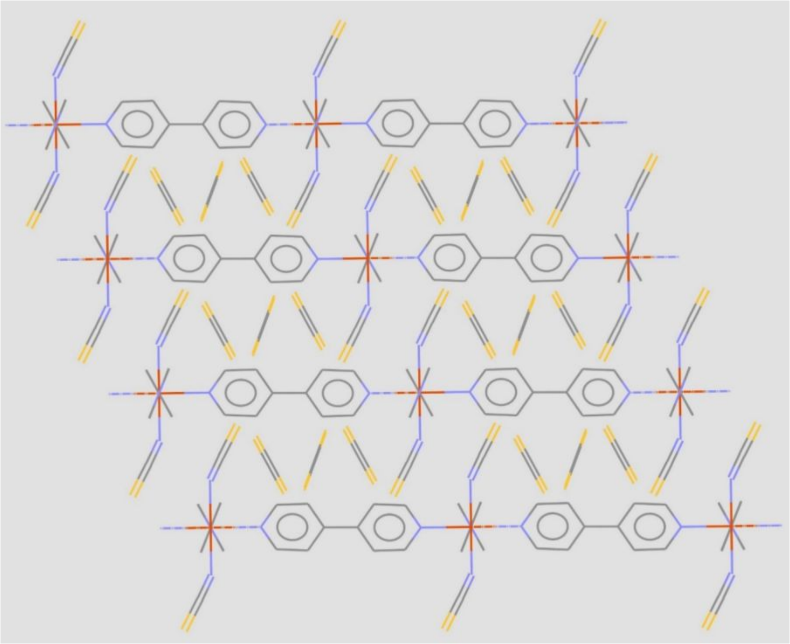

Figure S21. Layer packing fashion (viewed along ***b*** axis) of sql-1-Fe-NCS·3CS_2 (refcode: QAGXED).

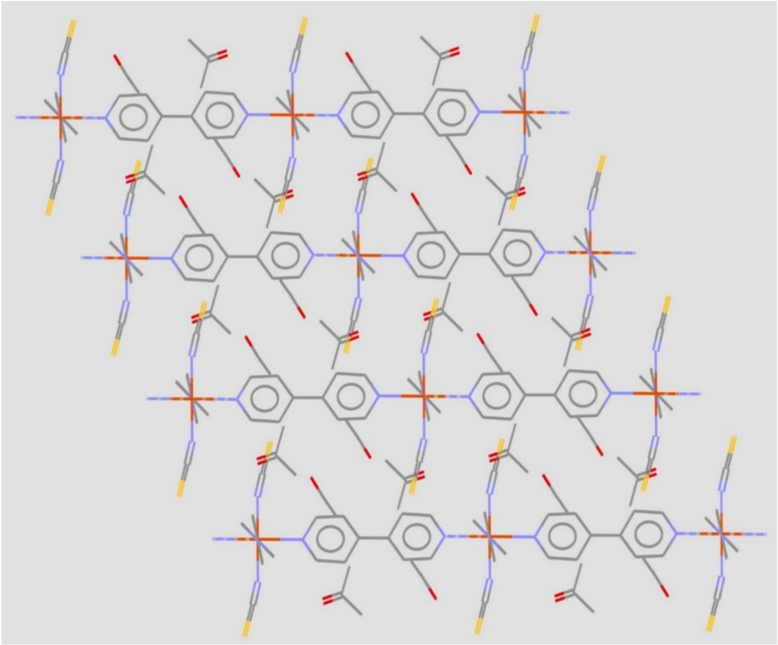

Figure S22. Layer packing fashion (viewed along ***b*** axis) of sql-1-Fe-NCS·4C_3H_6O (C_3H_6O = acetone, refcode: QAGWOM).

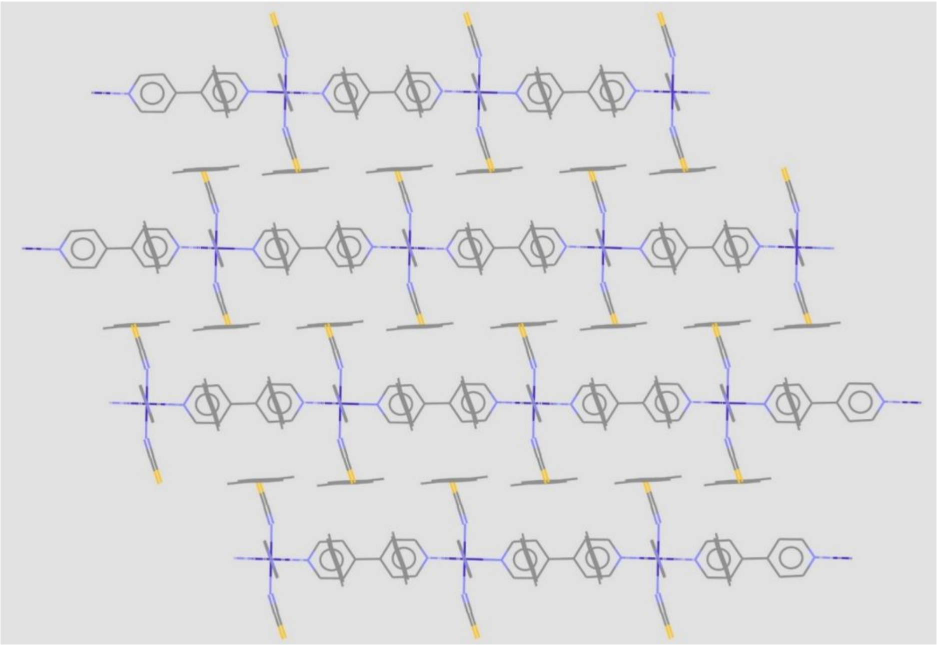

Figure S23. Layer packing fashion (viewed along ***b*** axis) of sql-1-Co-NCS·4PX (PX = *p*-xylene, refcode: KODFER).

Table S1. Crystallographic information of different phases of sql-1-Ni-NCS.

	**cp**	**lp (3CO$_2$)**	**lp (4C$_2$H$_{4/6}$)**	**ip (2C$_2$H$_{4/6}$)**	**lp (4C$_2$H$_2$)**
SG	*C2/c*	*C2/c*	*C2/c*	*C2/c*	*P2$_1$/n*
a/Å	12.156	12.601	13.383	13.652	10.118
b/Å	11.381	11.368	11.381	11.371	16.117
c/Å	16.646	19.563	22.356	20.903	10.065
β/º	100.43	93.41	105.56	101.32	104.21
V/Å^3	2264.9	2797.3	3280.2	3181.9	1590.9
Z	4	4	4	4	2

Table S2. Structural parameters of sql-1-NCS and its gas-loaded phases.

Switching CNs	$\angle_{Ni\text{-}Ni\text{-}Ni}$ [º]	$\angle_{Ni\text{-}N\text{-}CS}$ [º]	Torsion angle of bpy [º]	Interlayer separation [Å]
sql-1-Ni-NCS (ref 1)	76.4	169.5	54.7	4.5
sql-1-Ni-NCS·3CO$_2$ (ref 1)	90.0	162.1	63.6	5.4
sql-1-Ni-NCS·4C$_2$H$_{4/6}$	86.2	151.2	57.1	6.3
sql-1-Ni-NCS·2C$_2$H$_{4/6}$	89.5	140.8	82.0	6.2

Table S3. Crystallographic information of previously reported **sql** CNs with chemical formula $[M(bpy)_2(NCS)_2]\cdot xG$ (**sql-1-M-NCS·*x*G**) (**M** = Fe, Co, and Ni, **G** = guest).[4]

**sql-1-M-NCS**	**refcode**	**SG**	***a*/Å**	***b*/Å**	***c*/Å**	***β*/°**	**Z**	**V/Å^3**
sql-1-Ni-NCS	CIZFOG	*C2/c*	12.16	11.38	16.65	100.43	4	2264.9
sql-1-Ni-NCS·3CO_2	TAPLUV	*C2/c*	12.60	11.37	19.56	93.40	4	2797.3
sql-1-Ni-NCS·4C_2H_2	-	*P2₁/n*	10.12	16.12	10.07	104.21	2	1590.9
sql-1-Co-NCS	YUVROX	*C2/c*	12.25	11.42	16.55	100.17	4	2279.6
	YUVROX01	*C2/c*	12.10	11.40	16.54	99.94	4	2246.6
sql-1-Co-NCS·3CO_2	VIBQUV	*C2/c*	12.61	11.47	19.68	92.92	4	2841.7
sql-1-Co-NCS·2TFT	VIBQOP	*C2/c*	23.35	11.52	13.78	108.76	4	3510.4
sql-1-Co-NCS·2$C_4H_{10}O$	RINPUZ	*P2/c*	11.48	11.40	13.61	107.55	2	1698.1
sql-1-Co-NCS·2EB	KODFIV	*C2/c*	21.85	11.47	13.44	103.14	4	3280.4
sql-1-Co-NCS·4OX	KODDUF	*C2/c*	20.64	11.46	22.92	116.25	4	4860.8
sql-1-Co-NCS·4MX	KODFAN	*P2₁/n*	11.39	16.10	13.33	97.91	2	2422.0
sql-1-Co-NCS·4PX	KODFER	*C2/c*	23.61	11.48	20.22	117.37	4	4866.4
sql-1-Fe-NCS	TAPLIJ	*C2/c*	12.17	11.54	16.67	100.33	4	2303.7
sql-1-Fe-NCS·3CO_2	TAPLOP	*C2/c*	12.58	11.54	19.81	92.57	4	2871.6
sql-1-Fe-NCS·2S_8	ETOFAW	*C2/c*	21.25	11.59	16.46	105.75	4	3902.0
sql-1-Fe-NCS·2C_2HCl_3	QAGVUR	*C2/c*	22.51	11.55	12.36	103.30	4	3128.3
sql-1-Fe-NCS·2MB	QAGWAY	*C2/c*	21.33	11.58	12.99	99.35	4	3167.5
sql-1-Fe-NCS·2NB	QAGWEC	*C2/c*	22.47	11.61	13.21	103.08	4	3354.2
sql-1-Fe-NCS·2$C_4H_{10}O$	QAGWIG	*P2/c*	11.52	11.48	13.10	106.60	2	1660.4
sql-1-Fe-NCS·3CS_2	QAGXED	*P2/c*	11.53	11.54	12.75	110.83	2	1585.5
sql-1-Fe-NCS·3MeOH	QAGWUS	*C2/c*	16.05	16.61	12.60	120.58	4	2891.8
sql-1-Fe-NCS·2NM·MeOH	QAGXAZ	*C2/c*	16.42	16.38	12.55	120.21	4	2916.2
sql-1-Fe-NCS·4C_3H_6O	QAGWOM	*P2/c*	11.55	11.54	15.45	114.37	2	1875.7
sql-1-Fe-NCS·4$CHCl_3$	QAQTOT	*C2/c*	22.68	22.62	17.29	117.76	8	7849.7

Table S4. Structural parameters of different phases of ELM-11 (sql-1-Cu-BF_4).

**Switching CNs**	**SG**	***a*/Å**	***b*/Å**	***c*/Å**	***β*/°**	**Z**	**V/Å^3**	**Ref.**
ELM-11 (273 K)	*C2/c*	12.42	11.16	16.14	100.53	4	2200.5	5
ELM-11·2CO_2 (195 K)	*C2/c*	13.58	11.09	18.56	94.43	4	2785.8	5
ELM-11·2CO_2 (298 K)	*C2/c*	13.72	11.05	18.75	95.92	4	2829.4	5
ELM-11·3CO_2 (195 K)	*P2/c*	13.69	11.09	18.46	94.17	4	2794.9	6
ELM-11·6CO_2 (195 K)	*P1*	11.09	11.12	14.39	75.51	2	1713.8	6
ELM-11·2C_4H_{10} (273 K)	*C2/c*	20.18	11.00	15.09	105.73	4	3232.4	7

References

1. Wang, S.-Q.; Darwish, S.; Sensharma, D.; Zaworotko, M. J. Tuning the switching pressure in square lattice coordination networks by metal cation substitution. *Mater. Adv.* **2022,** *3* (2), 1240-1247.

2. Wang, S.-Q.; Mukherjee, S.; Zaworotko, M. J. Spiers Memorial Lecture: Coordination networks that switch between nonporous and porous structures: an emerging class of soft porous crystals. *Faraday Discuss.* **2021,** *231*, 9-50.

3. Wang, S.-Q.; Meng, X.-Q.; Vandichel, M.; Darwish, S.; Chang, Z.; Bu, X.-H.; Zaworotko, M. J. High Working Capacity Acetylene Storage at Ambient Temperature Enabled by a Switching Adsorbent Layered Material. *ACS Appl. Mater. Interfaces* **2021,** *13* (20), 23877-23883.

4. Wang, S.-Q.; Darwish, S.; Zaworotko, M. J. Adsorbate-dependent phase switching in the square lattice topology coordination network $[Ni(4,4'\text{-bipyridine})_2(NCS)_2]_n$. *Chem. Commun.* **2023**, *59* (5), 559-562.

5. Hiraide, S.; Tanaka, H.; Miyahara, M. T. Understanding gate adsorption behaviour of CO_2 on elastic layer-structured metal-organic framework-11. *Dalton Trans.* **2016,** *45* (10), 4193-4202.

6. Hiraide, S.; Tanaka, H.; Ishikawa, N.; Miyahara, M. T. Intrinsic Thermal Management Capabilities of Flexible Metal-Organic Frameworks for Carbon Dioxide Separation and Capture. *ACS Appl. Mater. Interfaces* **2017,** *9* (46), 41066-41077.

7. Bon, V.; Senkovska, I.; Wallacher, D.; Heerwig, A.; Klein, N.; Zizak, I.; Feyerherm, R.; Dudzik, E.; Kaskel, S. In situ monitoring of structural changes during the adsorption on

flexible porous coordination polymers by X-ray powder diffraction: Instrumentation and experimental results. *Microporous Mesoporous Mater.* **2014,** *188*, 190-195.
